# Supplementary material for: BMI prediction within a Korean population
Source: PeerJ. 2017 Jun 29;5:e3510. doi: 10.7717/peerj.3510 (PMC5493974; doi:10.7717/peerj.3510)
Supplement: Supplemental Information 1 [file peerj-05-3510-s001.docx]

**Supplementary Table 1. Clinical characteristics**

|  |  | Subjects | Age (Min-Max) | BMI (Min-Max) |
| --- | --- | --- | --- | --- |
| Total | All | 6,011 | 51.57 (40-69) | 24.58 (15.10-42.00) |
|  | Man | 2,903 | 51.07 (40-69) | 24.31 (15.10-36.10) |
|  | Woman | 3,108 | 52.04 (40-69) | 24.84 (16.10-42.00) |
|  |  |  |  |  |
| SNP selection set | All | 5,410 | 51.57 (40-69) | 24.58 (15.10-42.00) |
|  | Man | 2,613 | 51.05 (40-69) | 24.30 (15.10-36.10) |
|  | Woman | 2,797 | 52.06 (40-69) | 24.85 (16.10-42.00) |
|  |  |  |  |  |
| Final validation set | All | 601 | 51.54 (40-69) | 24.56 (16.40-34.60) |
|  | Man | 290 | 51.20 (40-69) | 24.40 (16.50-32.10) |
|  | Woman | 311 | 51.86 (40-69) | 24.71 (16.40-34.60) |

BMI, Body Mass Index;

**Supplementary Table 2. Results of regression analyses and allele information of selected SNPs in 10-fold cross-validation**

|  |  |  |  | *P*-Values | | | | | | | | | | |  | | Allele information | | | | |  | | Genotype Count with BMI average | | | |  | |  | |
| --- | --- | --- | --- | --- | --- | --- | --- | --- | --- | --- | --- | --- | --- | --- | --- | --- | --- | --- | --- | --- | --- | --- | --- | --- | --- | --- | --- | --- | --- | --- | --- |
| Markers | Gene | Location |  | Set1 | Set2 | Set3 | Set4 | Set5 | Set6 | Set7 | Set8 | Set9 | Set10 |  | | BMI increasing | | Minor | Major | MAF |  | | C/C  (BMI log) | | C/R  (BMI log) | R/R  (BMI log) |  | | LD (r^2^>0.8) | |  |
| *rs12566985* | *FPGT-TNNI3K* | 1:74536509 |  | 0.01 | 0.02 | 0.01 | 0.01 | 0.008 | 0.01 | 0.006 | 0.002 | 0.004 | 0.002 |  | | G | | A | G | 0.11 |  | | 3837 (3.197) | | 950  (3.188) | 81  (3.171) |  | | *-* | |  |
| *rs6545809* | *ADCY3* | 2:24903846 |  | 0.02 | 0.04 | 0.03 | 0.02 | 0.03 | 0.03 | 0.01 | 0.02 | 0.05 | 0.007 |  | | T | | T | C | 0.44 |  | | 1527 (3.191) | | 2397  (3.193) | 944  (3.204) |  | | *rs10182181* | |  |
| *rs2943634* | *-* | 2:226203364 |  | 0.002 | 0.0005 | 0.004 | 0.0008 | 0.003 | 0.0009 | 0.005 | 0.002 | 0.02 | 0.004 |  | | C | | A | C | 0.08 |  | | 4128 (3.197) | | 718  (3.180) | 22  (3.196) |  | | *-* | |  |
| *rs734597* | *-* | 6:50868566 |  | 0.03 | 0.04 | 0.04 | 0.04 | 0.02 | 0.03 | 0.02 | 0.01 | 0.04 | 0.05 |  | | A | | A | G | 0.19 |  | | 3200 (3.192) | | 1499  (3.200) | 169  (3.203) |  | | *rs987237* | |  |
| *rs11030104* | *BDNF* | 11:27662970 |  | 0.001 | 0.003 | 0.003 | 0.002 | 0.006 | 0.006 | 0.002 | 0.008 | 0.0005 | 0.004 |  | | A | | G | A | 0.45 |  | | 1472 (3.199) | | 2423  (3.196) | 973  (3.182) |  | | *-* | |  |
| *rs7988412* | *GTF3A* | 13:27426145 |  | 0.01 | 0.03 | 0.02 | 0.009 | 0.006 | 0.008 | 0.004 | 0.005 | 0.02 | 0.01 |  | | T | | T | C | 0.13 |  | | 3634 (3.192) | | 1159  (3.203) | 75  (3.195) |  | | *rs12016871* | |  |
| *rs2241423* | *MAP2K5* | 15:67794500 |  | 0.01 | 0.01 | 0.009 | 0.01 | 0.009 | 0.007 | 0.01 | 0.006 | 0.04 | 0.03 |  | | G | | G | A | 0.37 |  | | 1945 (3.191) | | 2261  (3.193) | 662  (3.208) |  | | *-* | |  |
| *rs7202116* | *FTO* | 16:53787703 |  | 0.003 | 0.006 | 0.001 | 0.001 | 0.003 | 0.004 | 0.006 | 0.003 | 0.0005 | 6.54x10^-5^ |  | | G | | G | A | 0.13 |  | | 3718 (3.191) | | 1058  (3.206) | 92  (3.198) |  | | *-* | |  |
| *rs6567160* | *-* | 18:60161902 |  | 0.002 | 0.006 | 0.01 | 0.002 | 0.008 | 0.006 | 0.002 | 0.01 | 0.009 | 0.004 |  | | C | | C | T | 0.24 |  | | 2783 (3.190) | | 1791  (3.198) | 294  (3.213) |  | | *-* | |  |
| *rs574367* | *-* | 1:176139833 |  | 0.008 | 0.04 | 0.03 | 0.01 | - | 0.02 | 0.03 | 0.009 | 0.04 | - |  | | T | | T | G | 0.24 |  | | 2784 (3.191) | | 1797  (3.199) | 287  (3.204) |  | | *-* | |  |
| *rs12286929* | *-* | 11:115151684 |  | 0.03 | - | - | - | 0.03 | 0.02 | 0.007 | - | - | - |  | | G | | G | A | 0.23 |  | | 2907 (3.191) | | 1720  (3.198) | 241  (3.204) |  | | *-* | |  |
| *rs12679556* | *-* | 8:71601993 |  | - | 0.04 | 0.05 | 0.01 | 0.03 | 0.04 | 0.04 | 0.03 | 0.05 | 0.03 |  | | G | | T | G | 0.24 |  | | 2776 (3.198) | | 1802  (3.191) | 290  (3.188) |  | | *-* | |  |
| *rs13072536* | *ITIH4* | 3:52827195 |  | - | - | 0.05 | 0.03 | - | 0.03 | - | - | - | - |  | | T | | T | A | 0.37 |  | | 1933  (3.19) | | 2273  (3.197) | 662  (3.199) |  | | *rs2535633* | |  |
| *rs9295474* | *CDKAL1* | 6:20652486 |  | - | - | 0.03 | 0.04 | - | 0.03 | - | - | - | 0.01 |  | | C | | G | C | 0.48 |  | | 1339 (3.199) | | 2412  (3.195) | 1117  (3.188) |  | | *rs2206734* | |  |
| *rs6090583* | *EYA2* | 20:46930192 |  | - | - | 0.02 | - | - | - | - | - | - | - |  | | G | | G | A | 0.18 |  | | 3315 (3.192) | | 1401  (3.200) | 152  (3.204) |  | | *-* | |  |
| *rs7138803* | *-* | 12:49853685 |  | - | - | 0.03 | 0.01 | - | - | - | 0.02 | - | - |  | | A | | A | G | 0.27 |  | | 2571 (3.192) | | 1918  (3.196) | 379  (3.205) |  | | *-* | |  |
| *rs12463617* | *-* | 2:629244 |  | - | - | - | 0.03 | - | - | - | - | - | - |  | | C | | A | C | 0.09 |  | | 4037 (3.196) | | 791  (3.186) | 40  (3.186) |  | | *-* | |  |
| *rs7607886* | *-* | 2:164653069 |  | - | - | - | 0.05 | - | - | - | - | - | - |  | | A | | A | G | 0.05 |  | | 4366 (3.194) | | 490  (3.203) | 12  (3.207) |  | | *rs12692738* | |  |
| *rs13179048* | *LOC-101929710* | 5:96207022 |  | - | - | - | 0.04 | - | - | - | - | 0.05 | 0.04 |  | | A | | A | C | 0.30 |  | | 2444 (3.192) | | 1974  (3.196) | 450  (3.204) |  | | *-* | |  |
| *rs6548238* | *-* | 2:634905 |  | - | - | - | - | 0.04 | 0.04 | 0.03 | - | - | - |  | | C | | T | C | 0.09 |  | | 4024 (3.200) | | 812  (3.186) | 33  (3.179) |  | | *-* | |  |
| *rs3736594* | *MRPL33* | 2:27772914 |  | - | - | - | - | - | - | 0.04 | 0.03 | 0.03 | - |  | | C | | C | A | 0.41 |  | | 1676 (3.191) | | 2396  (3.196) | 797  (3.201) |  | | *-* | |  |
| *rs12940622* | *RPTOR* | 17:80641771 |  | - | - | - | - | - | - | 0.04 | - | 0.04 | 0.008 |  | | G | | A | G | 0.33 |  | | 2213 (3.200) | | 2127  (3.190) | 529  (3.193) |  | | *-* | |  |
| *rs4256980* | *TRIM66* | 11:8652392 |  | - | - | - | - | - | - | - | 0.04 | - | - |  | | G | | G | C | 0.38 |  | | 1875 (3.191) | | 2307  (3.196) | 687  (3.201) |  | | *-* | |  |
| *rs6456368* | *CDKAL1* | 6:20659575 |  | - | - | - | - | - | - | - | - | 0.008 | - |  | | T | | C | T | 0.48 |  | | 1349 (3.199) | | 2394  (3.195) | 1126  (3.186) |  | | *rs7766070* | |  |
| *rs12111351* | *CDKAL1* | 6:20724327 |  | - | - | - | - | - | - | - | - | 0.05 | - |  | | T | | T | G | 0.48 |  | | 1366 (3.190) | | 2378  (3.194) | 1125  (3.200) |  | | *rs2206734* | |  |
| *rs3817334* | *MTCH2* | 11:47629441 |  | - | - | - | - | - | - | - | - | 0.03 | - |  | | T | | T | C | 0.31 |  | | 2289 (3.191) | | 2102  (3.195) | 478  (3.205) |  | | *-* | |  |
| *rs2256332* | *ITIH4* | 3:52821849 |  | - | - | - | - | - | - | - | - | 0.05 | - |  | | A | | A | G | 0.44 |  | | 1562 (3.188) | | 2373  (3.198) | 934  (3.196) |  | | *rs2535633* | |  |
| *rs10510760* | *PBRM1* | 3:52616332 |  | - | - | - | - | - | - | - | - | - | 0.03 |  | | A | | A | G | 0.50 |  | | 1242 (3.189) | | 2412  (3.195) | 1215  (3.200) |  | | *rs13083798* | |  |

Gene name and location and position of the SNPs were obtained from the NCBI database. The *P*-values were calculated using logistic regression on the SNP selection set as well as on the additional re-constructed sets (10 sets). Hyphens (-) indicate that not significant. C/C, C/R, and R/R represent the homozygote of the major allele, and the heterozygote and homozygote of the minor allele, respectively. The SNPs in the LD column are those reported in previous GWAS and were used in the present study. BMI, Body Mass Index; MAF, Minor allele frequency; LD, Linkage Disequilibrium.
