# Supplementary figures and images for: BMI prediction within a Korean population
Source: PeerJ. 2017 Jun 29;5:e3510. doi: 10.7717/peerj.3510 (PMC5493974; doi:10.7717/peerj.3510)

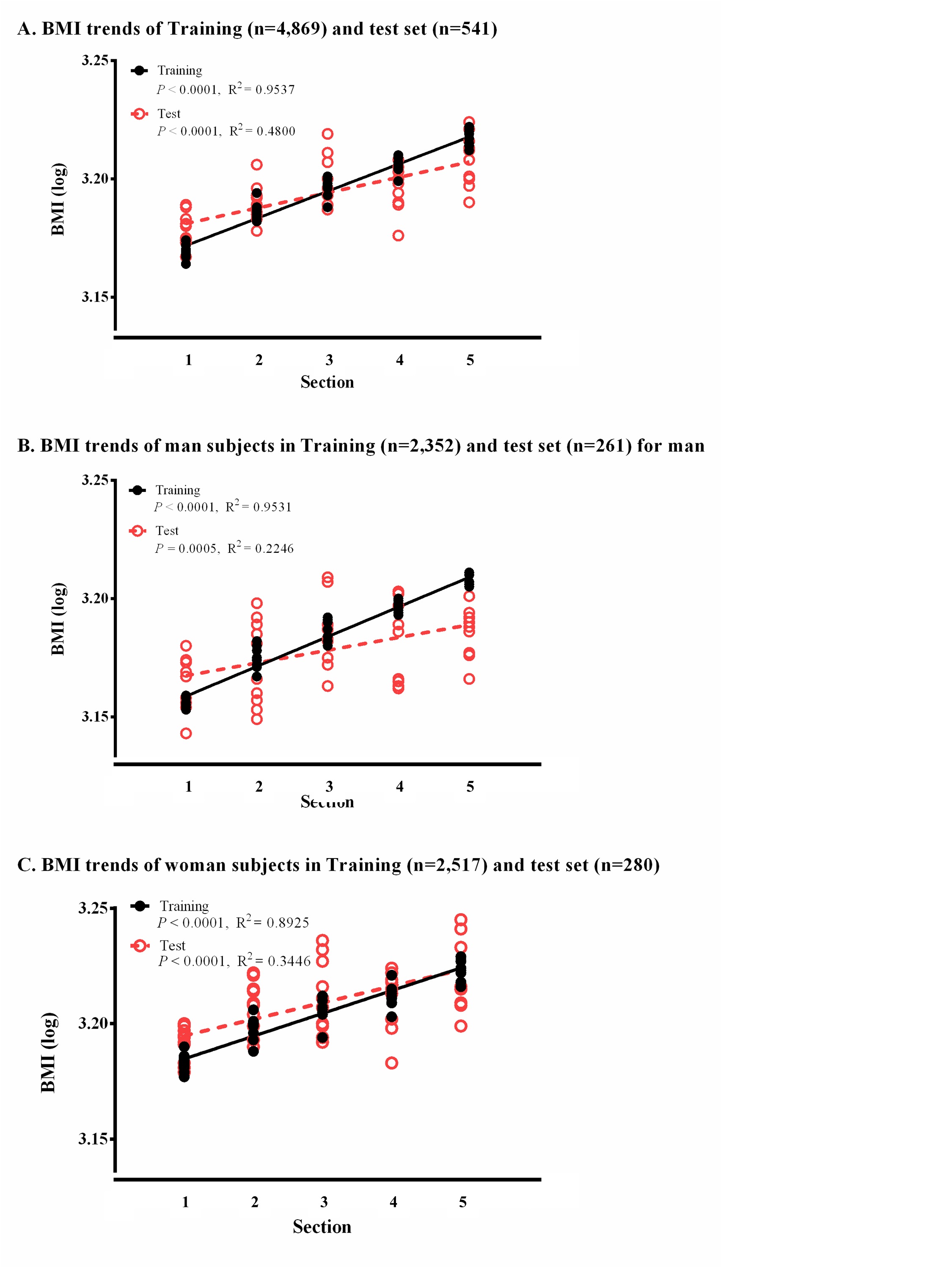

Supplement: Supplemental Information 2 — BMI trends of training sets and test sets. Each set was constructed using only 5,410 samples (4,869 for the training sets and 541 for the test sets). The overall P-values and R2 of each trend line are displayed. The black circle and red punctured circle represent the BMI values for the training set and test set, respectively, using 10-fold cross-validation. The black and red dashed lines are the standard curves for the complete training set and test set. (A) BMI trends of training set and test set using all samples. (B) BMI trends of training set and test set using male samples. (C) BMI trends of training set and test set using female samples. [file peerj-05-3510-s002.jpg]
